# Antibiotic Resistance and Typhoid

**DOI:** 10.1093/cid/ciy1111

**Published:** 2019-03-07

**Authors:** Zoe A Dyson, Elizabeth J Klemm, Sophie Palmer, Gordon Dougan

**Affiliations:** 1 Department of Medicine, University of Cambridge, Hinxton, Cambridge, United Kingdom; 2 Wellcome Sanger Institute, Wellcome Genome Campus, Hinxton, Cambridge, United Kingdom

**Keywords:** multiple drug resistance, MDR, *S*. Typhi, H58, typhoid

## Abstract

Multiple drug (antibiotic) resistance (MDR) has become a major threat to the treatment of typhoid and other infectious diseases. Since the 1970s, this threat has increased in *Salmonella enterica* serovar Typhi, driven in part by the emergence of successful genetic clades, such as haplotype H58, associated with the MDR phenotype. H58 *S*. Typhi can express multiple antibiotic resistance determinants while retaining the ability to efficiently transmit and persist within the human population. The recent identification of extensively drug resistant *S*. Typhi only highlights the dangers of ignoring this threat. Here we discuss the evolution of the *S*. Typhi MDR phenotype and consider options for management.

Since the introduction of antibiotics in the middle of the last century, there has been an increasing appreciation of the threat of antibiotic resistance [1, 2]. Following the first clinical use of penicillin, many other antibiotics have been introduced and, in each case, resistance has followed at some level. The threat of resistance has been compounded by a dramatic reduction in the rate of introduction of novel classes of antibiotics and an associated drop in investment in the area (https://amr-review.org). We are now in an era where the levels of circulating virulent, multiple drug-resistant (MDR) bacteria threatens healthcare efficacy globally. Typhoid treatment was one of the key areas that immediately benefited from antibiotic usage. This chronic infection with the potential for relapse, carriage, and complications was enormously challenging in terms of clinical management prior to antibiotics. Indeed, antibiotic treatment transformed the clinical management of the disease and the public’s perception of the threat the disease posed. Antibiotics, together with improved water treatment and better public health management, led to the control of the disease, at least in economically developed settings. However, typhoid remains in economically deprived areas where poverty and poor infrastructure persisted [3]. In these same resource-limited settings, the indiscriminate use of antibiotics has encouraged resistance and selected for virulent MDR clades. Thus, antibiotic resistance in typhoid is now a clinical and economic challenge.

*Salmonella enterica* serovar Typhi (*S.* Typhi) is a subtype of the Gram-negative enteric pathogen *Salmonella enterica*. Unlike many other *S. enterica* serovars, *S*. Typhi is a human restricted pathogen that, as far as we know, is propagated by human to human spread with no zoonotic reservoir and with a limited ability to survive longer term in the environment [4]. *S*. Typhi can persist in water and food contaminated with human fecal material, but there is no environmentally adapted stage of the bacterial life-cycle such as the formation of spores. The persistence of *S.* Typhi in human populations is influenced by clinically silent carriage within certain individuals (carriers) that can be infected for months and even years with periodic shedding of *S*. Typhi into the environment in contaminated feces. Thus, antibiotic usage can influence both acute typhoid disease and the carrier state. In both states the emergence of antibiotic resistance is theoretically possible. Unlike other enteric bacteria, genetic and phenotypic analysis (eg, through the controlled challenge of human volunteers) has indicated that *S*. Typhi is relatively poorly adapted for growth in the human intestine [5]. Indeed, many genes, for example, *shdA* associated with persistence in the intestine are inactivated in *S.* Typhi and are consequently known as pseudogenes [6]. *S*. Typhi also shows limited evidence of recombination with other bacteria, suggesting the global population of *S*. Typhi is relatively isolated compared to other enteric bacteria that live free in the intestine or environment [7]. This, theoretically, limits their relative ability to horizontally acquire genes, including those that are encoded on genetically mobile elements. Nevertheless, over the past 70 years, multiple antibiotic resistance in *S*. Typhi has emerged as a global threat.

## THE EARLY EMERGENCE OF ANTIBIOTIC RESISTANCE IN *S*. TYPHI

Shortly after the introduction of chloramphenicol for the treatment of typhoid, reports of resistance in *S*. Typhi began to emerge [8]. These tended to be reports of sporadic resistance associated with clinical failures and because new antibiotics were being introduced on a regular basis, particularly in the 1950–70s, this was largely regarded as a nuisance rather than a crisis. As *S*. Typhi has an unusual fastidious intracellular lifestyle in vivo, some antibiotics worked much better than others, and chloramphenicol became one of the “go to” antibiotics for this disease. The potential side effects of chloramphenicol dampened this enthusiasm to some extent, but cheaper antibiotics continued to be favored in resource-poor settings.

During the 1960s, resistance to 3 or more first-line (multiple) antibiotics including ampicillin, trimethoprim-sulfamethoxazole, and chloramphenicol, known as MDR, became more common, and MDR *S*. Typhi began to be reported more frequently. In 1972 a large MDR typhoid epidemic, with over 10 000 reported cases, occurred in Mexico City and other parts of Mexico [9, 10]. The epidemic *S*. Typhi commonly exhibited resistance to chloramphenicol, tetracycline, streptomycin, and sulphonamides and encoded an R factor plasmid that was transferable to *Escherichia coli*. Some *S*. Typhi isolates from the epidemic also encoded resistance to ampicillin and/or kanamycin, but fortunately these never became dominant. Eventually, this epidemic subsided and remained a local phenomenon confined largely within Mexico. Other sporadic cases of MDR *S*. Typhi were reported in different countries over the next 2 decades, but these also did not become dominant.

## MDR *S*. TYPHI BECAME A THREAT TO TREATMENT

During this period (1970–90) typhoid frequently occurred in regions with limited facilities for culturing *S*. Typhi and consequently the levels of MDR remained largely unreported and likely underappreciated. However, the Wellcome Trust and Oxford University established a Clinical Unit in Ho Chi Minh City in Vietnam in conjunction with the Vietnamese government, and a local program of typhoid surveillance involving blood culture and antibiotic susceptibility testing was established. It quickly became clear that MDR was associated with up to 90% of the typhoid cases in Ho Chi Minh City and in a rural study area in the Mekong Delta [11]. This, in part, explained a high level of clinical relapse, intestinal perforation, and poor response to treatment. Other reports coming in from elsewhere, including India [12], resulted in a rethink about the treatment of typhoid, and the use of fluoroquinolones in the form of ciprofloxacin was advocated [13]. To that point, the use of fluoroquinolones in children was not common due to reported side effects associated with joint/cartilage damage. Nevertheless, the present threat of MDR encouraged ethically supported trials of ciprofloxacin in the treatment of typhoid, and this was reported as being very effective with a low level of clinical relapse. However, within a few years of the introduction of ofloxacin treatment, *S*. Typhi exhibiting significant resistance to quinolones (nalidixic acid) and intermediate levels of resistance to ofloxacin were reported in the same region [14]. The targets of fluoroquinolones are the DNA gyrase subunits encoded by genes *gyrA* and *gyrB,* as well as DNA topoisomerase IV components *parC* and *parE*. “Signature” mutations including those at codon positions 83 and 87 in the *gyrA* gene sequence are associated with resistance. These signature mutations were identified in the nalidixic acid resistant derivatives of *S*. Typhi in Vietnam [15].

Over the next decade, the use of ciprofloxacin for the treatment of typhoid, and indeed other infections, became widespread across parts of Asia, and the reports of resistance began to increase in general frequency.

## THE EMERGENCE OF MDR CLADES OF *S*. TYPHI

As part of the studies on MDR *S*. Typhi in Vietnam, work was initiated to use molecular approaches including pulse field gel electrophoresis (PFGE) to characterize the *S*. Typhi and start to understand the population structure of the pathogen. An aim here was to try to track transmission and identify outbreaks in a region with a high level of endemic disease. It was quickly noted that the PFGE patterns of *S*. Typhi isolated before 1990 were diverse, but those isolated after this time onward became increasingly conserved as one PFGE type [16]. *S*. Typhi itself is highly clonal compared to many other pathogens, and this characteristic confounded the epidemiological investigations. This observation remained of interest, but the limitations of the techniques meant that this phenomenon remained unexplained. However, it was noted that all MDR *S*. Typhi harbored a large, transferrable R factor of incompatibility group IncHI1, the same group as the plasmid identified in Mexico in 1970 [17].

Over the next years, the interest in bacterial population structures increased as the technologies advanced. Techniques such as multi locus sequence typing (MLST) were invented, but they were not sufficiently discriminatory to provide further resolution of the population structure of *S*. Typhi, which is largely a single MLST type. Further advances into molecular typing using rare single nucleotide polymorphisms in the bacterial genome were eventually developed and applied to a global collection of *S*. Typhi [7]. These studies identified an emerging subtype, known as a haplotype, of *S*. Typhi designated H58 that was associated with both MDR and fluoroquinolone resistance signature mutations in *gyrA*. Importantly, *S*. Typhi H58 were found in many countries in Asia and were becoming more common in more recent isolates. The conserved PFGE MDR clade found in Vietnam was identified as haplotype H58. *S*. Typhi H58 frequently harbored MDR plasmids of IncHI1 type.

## WHOLE GENOME SEQUENCING OF *S*. TYPHI

In 2000, the first complete genome sequence of an MDR *S*. Typhi known as CT18 from Vietnam was reported [6]. *S*. Typhi CT18 is not H58, but it does harbor a classical *S*. Typhi-associated transferrable R plasmid named pHCM1. pHCM1 encodes resistance genes for all first-line antibiotics used for the treatment of typhoid fever and is highly related to R27, an IncHI1 plasmid that was first isolated in the 1960s from *S. enterica*. pHCM1 has acquired additional genes compared to R27, predominantly at 2 points with 18 genes encoding resistance to antibiotics and heavy metals. Multiple transposon-like and integrases/transposases are encoded around these 2 regions suggesting a recombination hotspot. Antibiotic resistance genes encoded on pHCM1 include *dhfr*1b (trimethoprim), *sul*2 (sulphonamide), *cat*I (chloramphenicol), *bla* (TEM-1; ampicillin), *tetA/tetC* (tetracyclines), and *strAB* (streptomycin). Further comparative analysis of IncHI1 plasmids showed that these plasmids were highly conserved in their broad framework but showed some variation at these two recombinatorial hotspots.

With the introduction of next generation sequencing the analysis of thousands of *S*. Typhi at the whole genome level rapidly became feasible and currently several thousand *S*. Typhi have been sequenced [18–21]. These data provide an enormous expanding database that has been used to define the global *S*. Typhi population structure and to analyze the antibiotic resistance signatures of individual isolates and clades. All currently circulating *S*. Typhi have a common ancestor and can be regarded as a monophyletic clade that entered the human population from an unknown source several thousand years ago. MDR isolates can be found around the phylogenetic tree, but over 50% of recent isolates belong to clade H58 with a significant proportion of these harboring MDR and fluoroquinolone resistance alleles [19].

However, clear evolutionary patterns can be detected. *S*. Typhi H58 isolates are widely distributed in Asia and the Middle East region. In addition, there have been at least 3 recent independent introductions of *S*. Typhi H58 into the African continent that have become established. These 3 “waves” are currently still present in Kenya, and the H58 introductions have spread across East Africa through several countries including Uganda, Tanzania, Zaire, and Malawi. *S*. Typhi H58 isolates have also entered Zimbabwe and South Africa but have not become dominant in West Africa. Interestingly, IncHI1 plasmids are not always present in newer *S*. Typhi isolates that express the MDR phenotype. Instead, regions of the composite transposon present on the IncHI1 plasmids have integrated into the *S*. Typhi chromosome, into at least 2 different genetic loci on multiple occasions. This was concerning in that chromosomally integrated DNA tends to be more stable and is less likely to be lost compared to a plasmid. Additionally, this highly fluid genetic region could act as a hot spot for sampling other genetic material taken up from the environment. Fluoroquinolone signature mutations are less common in *S*. Typhi from Africa than Asia, More recently a S. Typhi H58 “triple mutant” clade possessing 2 signature mutations in *gyrA* (codon 83 and 87) and a third in *parC* (codon 80) has been observed in Nepal as well as India and has been associated with a lack of fluoroquiolone efficacy [22, 23]. The reduced frequency of these mutations in Africa may be a reflection of the use of this class of antibiotics on the different continents [19].

Interestingly in West Africa, the evolution of MDR in *S*. Typhi appears to have taken a different path as H58 isolates have not become established there [24, 25]. Instead *S*. Typhi of haplotype H56 (3.1.1) that harbor a range of different R factor types, including some of IncHI1 type, have become established, for example, in Nigeria and Ghana. The genomic evidence indicates that several of these West African MDR clades have spread extensively in the region, crossing into multiple countries and causing sustained outbreaks. Similar clades have also been found in the Republic of Congo suggesting widespread typhoid in the region [26].

## THE EMERGENCE OF EXTENSIVELY RESISTANT *S*. TYPHI

**Figure 1.**
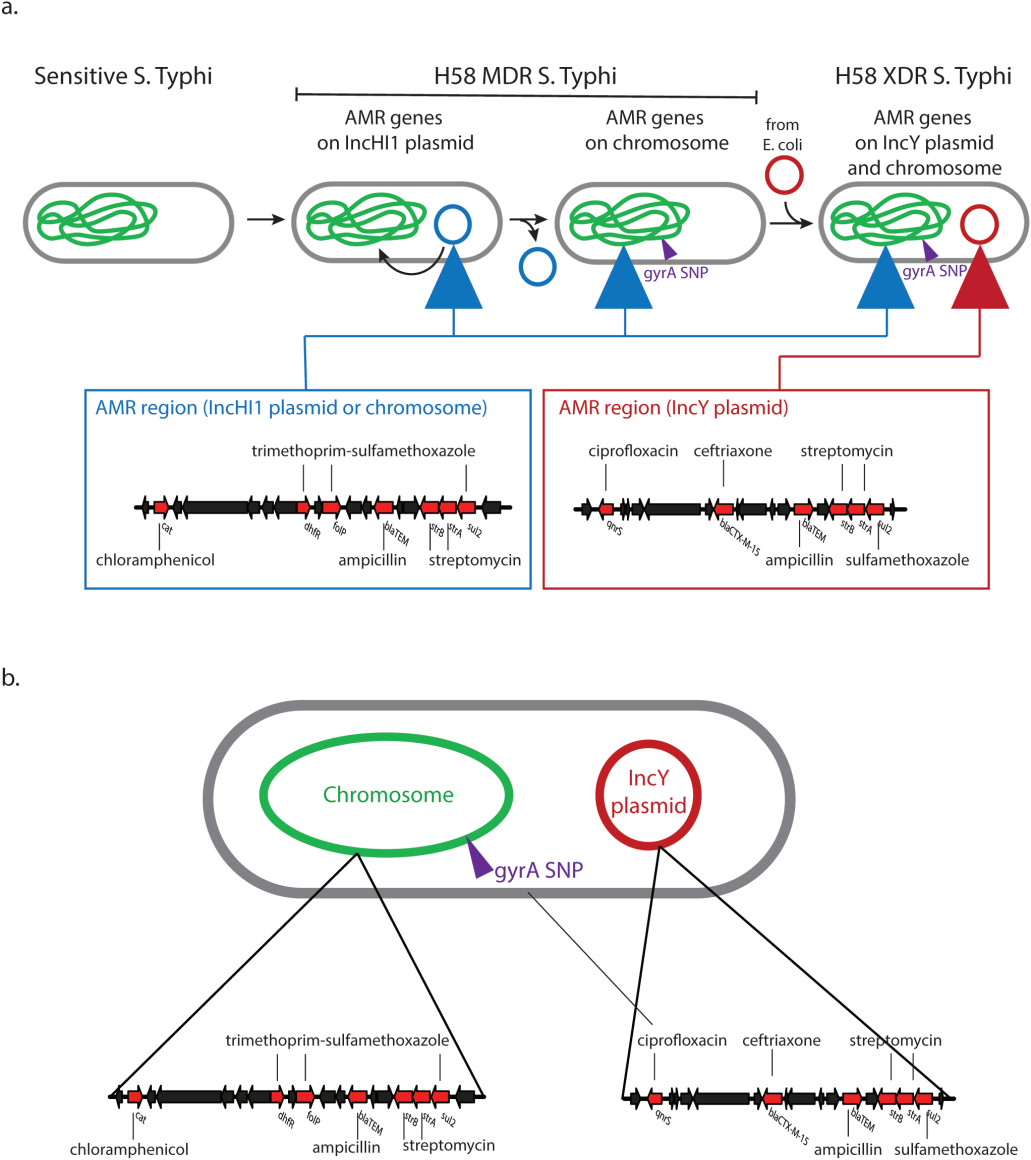
Outline genetic structure of XDR *S*. Typhi in Pakistan. *A*, Oval outlines represent individual bacteria harboring chromosome (wiggly, green) and plasmid (red and blue circles) elements. Red and blue triangles represent acquired resistance loci. Small purple triangles represent SNPs in the chromosome associated with resistance. *B*, Detailed structures with color codes as per *A* of the different acquired resistance elements on plasmid and chromosome. Abbreviations: AMR, antimicrobial resistance; MDR, multiple drug resistance; SNP, single nucleotide polymorphism; XDR, extensively drug resistant.

In 2017, reports emerged of a large outbreak in Sindh, Pakistan, of a *S*. Typhi expressing resistance to classical first-line antibiotics (chloramphenicol, ampicillin, and trimethoprim-sulfamethoxazole), as well as to both fluoroquinolones and third-generation cephalosporins. These infections can only be resolved with azithromycin, carbapenems (meropenem) and tigecycline (Table 1). Because only one of these remaining antibiotics can be administered orally, this presents a difficult and costly treatment practice, which is especially challenging in resource-limited settings. This outbreak stimulated an emergency response involving public health management and a typhoid vaccination campaign. The isolates were eventually classified as being “extensively drug resistant”[XDR] [27, 28]. Whole-genome sequencing of representative XDR isolates confirmed that this was indeed an outbreak involving a highly conserved subclade of the H58 haplotype. Subsequent genome analysis showed the clade harbored a very large pool of resistance determinants, with the chromosomally integrated composite resistance loci and an additional novel IncY plasmid encoding a *bla*_CTX-M-15_ extended-spectrum β-lactamase gene and a *qnrS* fluoroquinolone resistance gene (Figure 1). It is concerning that the *bla*_CTX-M-15_ and *qnrS* are encoded on the same plasmid raising the potential that treatment with one of the target antibiotic may favor the retention of both genes. The IncY plasmid was highly homologous to a similar plasmid group reported widely in other enteric bacteria around the globe, suggesting that the IncY plasmid may have been acquired by conjugation from an *E. coli* cohabiting the intestine of an individual [27]. Further genetic analysis showed that the H58 subclade associated with XDR was quite widely spread in Pakistan and other countries in the Middle East before the IncY plasmid was acquired in Sindh. Worryingly, the same XDR *S*. Typhi clade was isolated from a traveler returning from Pakistan to the United Kingdom and United States, indicating the clade is capable of intercontinental spread ^[27]^. There have been other reports of sporadic cases of XDR *S*. Typhi involving non-H58 isolates and different plasmids [28].

**Table 1.** Treatment Options for Different Resistance Classes of *S*. Typhi

Antibiotic	Route of Administration	MDR	Pakistan XDR
Chloramphenicol	Oral, intravenous	X	X
Co-trimoxazole	Oral, intravenous	X	X
Ampicillin	Oral, intramuscular, intravenous	X	X
Ciprofloxacin	Oral, intravenous		X
Ceftriaxone	Intramuscular, intravenous		X
Azithromycin	Oral		
Meropenem	Intravenous		
Tigecycline	Intravenous		

Abbreviations: MDR, multiple drug resistance; XDR, extensively drug resistant.

## WHAT NEXT?

These recent developments involving XDR isolates highlights the evolving threat of antibiotic resistance in *S*. Typhi and the value of antibiotic susceptibility testing linked to genomic analysis. Thus, we need to develop further low-cost diagnostic tools and kits that can be used in the field to rapidly detect such strains. Such tools should be linked to an open access, global database of whole genome-based sequences of *S*. Typhi. Such databases do exist in various DNA sequence repositories, but they are not readily accessible to groups or organizations not well trained in the genomic sciences. We need to develop simple software that can be used to rapidly select diagnostic signatures from these data that can be quickly used to modify and update diagnostic tools. Isothermal polymerase chain reaction technologies are being developed that are relatively inexpensive and can work on simple platforms such as dipsticks and these will be vital, potentially along with better serological and culture-based technologies for typhoid. These diagnostic approaches will be used in conjunction with the implementation of better public health management approaches including vaccine campaigns to bring typhoid under control in endemic regions. However, it should be noted that vaccination would likely not specifically target resistant oversensitive isolates, but it would at least reduce the overall typhoid disease burden.

Finally, it should be noted that the incidence of MDR in typhoid is variable over time. As antibiotic treatment practices change, the selective pressure profile is altered, and the incidence of MDR typhoid has been observed to increase and decrease over time in a particular geographical region. This suggests that selection is playing a significant role in the maintenance of the resistance phenotype and that better antibiotic stewardship could have a beneficial impact.

## Notes

***Financial support.*** This publication is based on research funded in part by a grant from the Bill & Melinda Gates Foundation [OPP1151153]. In addition, this work was supported by The Wellcome Trust (grants STRATAA, 106158 and 098051) and by the NIHR (Cambridge Biomedical Research Centre at the Cambridge University Hospitals NHS Foundation Trust).

***Supplement sponsorship.*** This supplement is sponsored by the Center for Vaccine Development and Global Health (CVD) at the University of Maryland School of Medicine.

***Potential conflicts of interest.*** All authors: No reported conflicts. All authors have submitted the ICMJE Form for Disclosure of Potential Conflicts of Interest. Conflicts that the editors consider relevant to the content of the manuscript have been disclosed.
